# Carotid artery wall shear stress is independently correlated with renal function in the elderly

**DOI:** 10.18632/oncotarget.23825

**Published:** 2018-01-02

**Authors:** Yuqi Guo, Fang Wei, Juan Wang, Yingxin Zhao, Shangwen Sun, Hua Zhang, Zhendong Liu

**Affiliations:** ^1^ Cardio-Cerebrovascular Control and Research Center, Institute of Basic Medicine, Shandong Academy of Medical Sciences, Jinan, Shandong 250062, China; ^2^ Department of Cardiology, Jinan Central Hospital Affiliated to Shandong University, Jinan, Shandong 250013, China; ^3^ Department of Cardiology, The Second Hospital of Shandong University, Jinan, Shandong 250000, China

**Keywords:** hemodynamic, wall shear stress, endothelial function, renal function, chronic kidney disease

## Abstract

Hemodynamic has increasingly been regarded as an important factor of renal function. However, the relationship between carotid artery wall shear stress (WSS) and renal function is not clarified. To investigate the relationship between carotid WSS and renal function, we recruited 761 older subjects aged 60 years and over from community-dwelling in the Shandong area, China. Carotid WSS, endothelial function, and estimated glomerular filtration rate (eGFR) were assessed in all subjects. Subjects were grouped by the interquartile of the carotid artery mean WSS. We found that the eGFRs derived from serum creatinine and/or cystatin C using three CKD-EPI equations were significantly higher and albumin/creatinine ratio was lower in the higher interquartile groups than in the lower interquartile groups (*P* <0.05). The mean WSS was independently correlated with eGFRs even after adjustment for confounders. Similar findings were found between carotid artery peak WSS and eGFRs and albumin/creatinine ratio. In addition, we found that endothelial function was strongly related to carotid WSS and renal function after adjustment for confounders. In conclusion, there is an independent correlation of carotid WSS with renal function in the elderly. The local rheologic forces may play an important role in renal function changing. The correlation may be mediated by regulation of endothelial function.

## INTRODUCTION

Chronic kidney disease (CKD), characterized by reduced glomerular filtration rate (GFR) or abnormal urinary albumin excretion [1, 2], is regarded as a part of a subclinical and generalized atherothrombosis [3]. It is seemed as a silent killer due to very mild symptoms and going unnoticed for a long time. Epidemiological investigation showed that the prevalence of CKD is approximately from 8% to 16% in various regions [4–8]. Now CKD is increasingly common in both developed and developing countries and rapidly becoming a global public health concern.

CKD is deemed to share many traditional atherosclerosis risk factors including diabetes, hypertension, and smoking with cardiovascular diseases [9–11]. However, these traditional risk factors cannot fully account for this high prevalence of CKD. Other non-traditional risk factors such as endothelial dysfunction, hemodynamic, and chronic inflammation have increasingly been studied [3, 12, 13].

It is known that the endothelium is highly distributed among all organ systems [14]. Due to the specialized and variable nature of the endothelium, the kidney tends to be a target organ in systemic disease [15]. It has been shown that renal function is closely related to endothelial dysfunction in an elderly general population with mildly impaired renal function [16].

Wall shear stress (WSS), also called endothelial shear stress, is one of the most important hemodynamic forces that are exerted by circulating blood on the endothelial cell layer of the inner vessel wall [17, 18]. It represents the tangential force per unit area exerted by flowing blood on the endothelial surface of the vascular wall and plays an important role in vascular remodeling and atherogenesis [19, 20]. Different WSSs are thought to have different effects on vascular activities [20, 21]. Generally, low or oscillatory WSS is regarded as a reliable indicator for vascular endothelial dysfunction [17–21].

Some studies [22, 23] have investigated the correlation renal function with either carotid artery WSS or brachial artery WSS in recent decades. However, the findings were inconsistencies. The possible relationship between local WSS and renal function has not been fully explored and illuminated although there are some studies made lots of efforts [22–25].

As a well established “observation window” for systemic structure and arterial function in humans and supplies a very precise regulation of blood flow [26], the common carotid artery (CCA) WSS may also represent the overall hemodynamic condition of the renal vessels [22, 25]. We hypothesized that carotid WSS is independently correlated with renal function in the elderly. And, in some extent, carotid WSS may be an important predictive factor of mildly impaired renal function. The main goal of the present study was to investigate and illuminate this relationship.

## RESULTS

### Demographic and clinical characteristics

The demographic and clinical characteristics of all subjects are summarized in Table 1. The interquartile of mean WSS was <0.85, 0.85-1.06, 1.07-1.26, and >1.26 Pa, and peak WSS was <1.49, 1.49-1.82, 1.83-2.15, and >2.15 Pa.

**Table 1:** Clinical and biochemical characteristics of total participants

Clinical parameters	Value
Age, years	70.70±6.15
Sex, Female:Male	380:381
Systolic blood pressure, mm Hg	141.33±13.72
Diastolic blood pressure, mm Hg	69.73±7.05
Body mass index, kg/m^2^	24.31±2.86
Biochemical parameters	
Total cholesterol, mmol/L	4.57±0.66
Triglycerides, mmol/L	1.45±0.34
High-density lipoprotein cholesterol, mmol/L	1.14±0.18
Low- density lipoprotein cholesterol, mmol/L	2.77±0.63
Fasting plasma glucose, mmol/L	5.303±0.91
Creatinine, mg/dl	0.78 (0.66, 0.91)
Cystatin C, mg/L	093±0.21
Urinary albumin, mg/L	1.26 (0.81, 1.92)
Covariates	
Smoking, *n* (%)	178 (23.39)
Alcohol intake, *n* (%)	311 (40.87)
Hypertension, *n* (%)	489 (64.26)
Antihypertensive medication, *n* (%)	445 (91.00)
Diabetes, *n* (%)	93 (12.22)
Lowering glucose medication, *n* (%)	87 (93.55)
Dyslipidemia, *n* (%)	220 (28.91)
Anti-dyslipidemia medication, *n* (%)	108 (19.09)
Carotid plaque, *n* (%)	262 (34.43)
Carotid wall shear stress	
Carotid mean wall shear stress, Pa	1.06±0.28
Carotid peak wall shear stress, Pa	1.84±0.45
Endothelial function and inflammatory parameters	
Brachial artery flow-mediated dilation, %	11.84±3.03
Nitric oxide, μmol/L	63.08±14.50
Endothelin-1, pg/ml	42.28±9.09
Intercellular adhesion molecule-1, ng/ml	140.39±35.30
Vascular cell adhesion molecule-1, ng/ml	613.58 (544.56, 699.06)
Renal function parameters	
eGFR_Cr_, ml·min^-1^·1.73m^-2^	82.52±17.48
eGFR_CysC_, ml·min^-1^·1.73m^-2^	81.48±20.77
eGFR_Cr-CysC_, ml·min^-1^·1.73m^-2^	83.17±18.96
Albumin/creatinine ratio, mg/g	17.59±7.25

Continuous data are expressed as mean ± standard deviation or as median with interquartile range depending on the normality of the data. Categorical data are expressed as numbers (percentages). eGFR_Cr_ indicates estimated glomerular filtration rate base on creatinine; eGFR_CysC_, estimated glomerular filtration rate base on cystatin C; eGFR_Cr-CysC_, estimated glomerular filtration rate base on creatinine and cystatin C.

Table 2 shows the demographic and clinical characteristics of subjects grouped by interquartile of mean WSS. The brachial artery flow-mediated dilation (FMD) and serum nitric oxide (NO) significantly increased, and the serum endothelin-1 (ET-1) and intercellular adhesion molecule-1 (ICAM-1) decreased from Q1 to Q4 according to the respective group. The differences were significant between any two groups (all *P* <0.05). The vascular cell adhesion molecule-1 (VCAM-1) was markedly lower in Q4 than in Q1, Q2, and Q3, and lower in Q2 and Q3 than in Q1 (all *P* <0.05).

**Table 2:** Clinical and biochemical characteristics of participants grouped by the interquartile of carotid mean wall shear stress

	Q1 (*n*=192)	Q2 (*n*=189)	Q3 (*n*=190)	Q4 (*n*=190)	*P* value
Clinical parameters					
Age, years	72.33±6.29	71.91±6.07	70.15±6.10^*,†^	68.41±5.33^*,†^	<0.001
Sex, Male:Female	95:97	99:90	93:97	94:96	0.907
BMI, kg/m^2^	24.73±2.80	24.41±2.90	24.23±3.01	23.89±2.68^*^	0.037
SBP, mm Hg	146.28±13.49	142.67±12.74^*^	140.86±13.39^*^	135.47±13.07^*,†,‡^	<0.001
DBP, mm Hg	69.82±7.29	70.65±6.75	68.97±7.12	69.48±6.97	0.129
Biochemical parameters					
TCHO, mmol/L	4.61±0.68	4.60±0.65	4.57±0.64	4.51±0.66	0.509
TG, mmol/L	1.46±0.38	1.43±0.33	1.46±0.33	1.45±0.34	0.852
HDL-c, mmol/L	1.12±0.17	1.11±0.18	1.14±0.18	1.16±0.19	0.246
LDL-c, mmol/L	2.82±0.67	2.81±0.63	2.76±0.61	2.70±0.62	0.208
FPG, mmol/L	5.38±0.95	5.44±1.02	5.30±0.92	5.18±0.74^*^	0.033
Cr, mg/dl	0.85 (0.71, 1.06)	0.82 (0.70, 0.96)^*^	0.78 (0.64, 0.89)^*,†^	0.8 (0.60, 0.79)^*,†,‡^	<0.001
Cys C, mg/L	1.03±0.17	0.98±0.21^*^	0.93±0.19^*^	0.78±0.18^*,†,‡^	<0.001
UA, mg/L	1.92 (1.38, 2.82)	1.42 (0.96, 2.07)^*^	1.13 (0.80, 1.72)^*,†^	0.78 (0.54, 1.05)^*,†,‡^	<0.001
Covariates					
Current smoker, *n* (%)	36 (18.75)	50 (26.46)	46 (24.21)	46 (24.21)	0.329
Alcohol consumption, *n* (%)	54 (28.13)	83 (43.92)^*^	83 (43.68)^*^	89 (46.84)^*^	0.001
Hypertension, *n* (%)	146 (76.04)	133 (70.37)	117 (61.58)^*^	93 (48.95)^*,†,‡^	<0.001
Antihypertension, *n* (%)	133 (91.10)	121 (90.98)	106 (90.60)	84 (90.32)	0.997
Diabetes, *n* (%)	21 (10.94)	25 (13.23)	26 (13.68)	21 (11.05)	0.855
Antidiabetes, *n* (%)	20 (95.24)	22 (88.00)	25 (96.15)	20 (95.24)	0.622
Dyslipidemia, *n* (%)	58 (30.21)	49 (25.93)	53 (27.89)	60 (31.58)	0.630
Anti-dyslipidemia, *n* (%)	31 (53.45)	26 (53.06)	24 (45.28)	27 (45.00)	0.692
Carotid plaque, *n* (%)	112 (58.33)	68 (35.98) ^*^	63 (33.16) ^*^	19 (10.00) ^*,†,‡^	<0.001
Endothelial function parameters					
Brachial artery FMD, %	9.56±2.44	11.01±2.47^*^	12.44±2.57^*,†^	14.39±2.30^*,†,‡^	<0.001
NO, μmol/L	51.39±11.93	60.17±10.70^*^	65.14±11.84^*,†^	75.71±11.63^*,†,‡^	<0.001
ET-1, pg/ml	49.06±8.24	44.13±8.20^*^	40.58±7.24^*,†^	35.27±6.46^*,†,‡^	<0.001
ICAM-1, ng/ml	164.00±37.21	143.59±33.03^*^	132.31±29.49^*,†^	121.45±25.58^*,†,‡^	<0.001
VCAM-1, ng/ml	674.94 (605.92, 766.98)	636.59 (582.91, 708.31)^*^	613.58 (550.73, 699.30)^*^	544.56 (480.19, 613.58)^*,†,‡^	<0.001

Data are mean±standard deviation or number (percentage). Q1 indicates the first interquartile; Q2, the second interquartile; Q3, the third interquartile; Q4, the forth interquartile; BMI, body mass index; SBP, systolic blood pressure; DBP, diastolic blood pressure; TCHO, total cholesterol; TG, triglycerides; HDL-c, high-density lipoprotein cholesterol; LDL-c, low-density lipoprotein cholesterol; Cr, creatinine; Cys C, cystatin C; UA, Urinary albumin; FMD, flow-mediated dilation; NO, nitric oxide; ET-1, endothelin-1; ICAM-1, intercellular adhesion molecule-1; VCAM-1, vascular cell adhesion molecule-1. ^*^P <0.05, as compared to Q1; ^†^*P* <0.05, as compared to Q2; ^‡^*P* <0.05, as compared to Q3.

Supplementary Table 1 shows the demographic and clinical characteristics of subjects grouped by interquartile of peak WSS. The FMD significantly increased from Q1 to Q4 according to the respective group, and differences were significant between any two groups (all *P* <0.05). There was an increasing trend in NO, and decreasing trends in ET-1, ICAM-1, and VCAM-1 from Q1 to Q4. Compared with Q1, Q2, and Q3, the NO was significantly higher, and the ET-1, ICAM-1, and VCAM-1 were lower in Q4 (all *P* <0.05).

### Effect of mean WSS on renal function

Table 3 depicts the details of the estimated glomerular filtration rate base on creatinine (eGFR_Cr_), estimated glomerular filtration rate base on cystatin C (eGFR_CysC_), estimated glomerular filtration rate base on creatinine and cystatin C (eGFR_Cr-CysC_), and albumin/creatinine ratio (ACR) in the four groups classified by the interquartile of the mean WSS. The eGFR_Cr_, eGFR_CysC_, and eGFR_Cr-CysC_ significantly increased, and ACR decreased from Q1 to Q4, and difference were significant between any two groups (all *P* <0.05).

**Table 3:** Estimated glomerular filtration rates and albumin/creatinine ratio of participants grouped by the interquartile of carotid mean wall shear stress

	Q1 (*n*=192)	Q2 (*n*=189)	Q3 (*n*=190)	Q4 (*n*=190)	*P* value
eGFR_Cr_, ml·min^-1^·1.73m^-2^	72.37±18.40	79.67±15.88^*^	84.74±15.67^*,†^	93.39±12.37^*,†,‡^	<0.001
eGFR_CysC_, ml·min^-1^·1.73m^-2^	70.04±16.39	76.74±19.42^*^	82.31±19.21^*^	96.91±18.00^*,†,‡^	<0.001
eGFR_Cr-CysC_, ml·min^-1^·1.73m^-2^	71.38±16.66	78.87±16.04^*^	84.67±16.77^*,†^	97.87±14.96^*,†,‡^	<0.001
ACR, mg/g	23.04±6.93	18.69±6.56^*^	16.36±6.11^*,†^	12.22±4.54^*,†,‡^	<0.001

Data are mean±standard deviation. Q1 indicates the first interquartile; Q2, the second interquartile; Q3, the third interquartile; Q4, the forth interquartile; eGFR_Cr_, estimated glomerular filtration rate base on creatinine; eGFR_CysC_, estimated glomerular filtration rate base on cystatin C; eGFR_Cr-CysC_, estimated glomerular filtration rate base on creatinine and cystatin C; ACR, albumin/creatinine ratio. ^*^P <0.05, as compared to Q1; ^†^*P* <0.05, as compared to Q2; ^‡^*P* <0.05, as compared to Q3.

**Figure 1:**
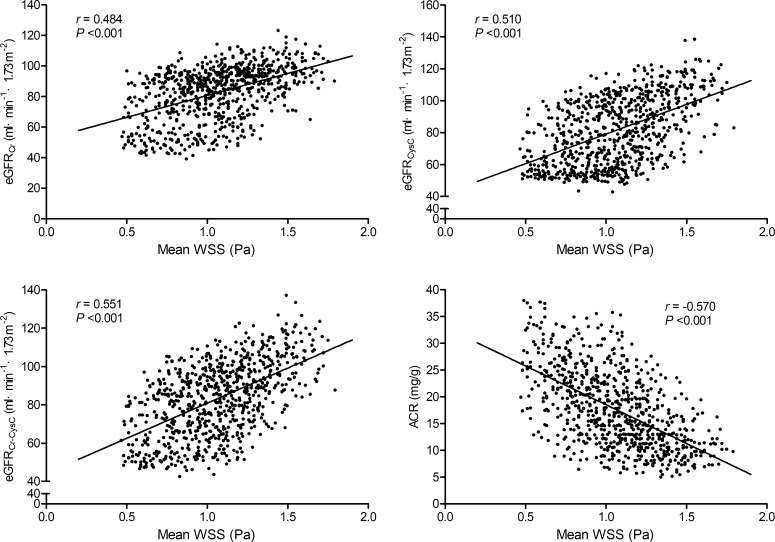
Correlations of carotid mean wall shear stress with the eGFR_Cr_, eGFR_CysC_, eGFR_Cr-CysC_, and ACR WSS indicates wall shear stress; eGFR_Cr_, estimated glomerular filtration rate base on creatinine; eGFR_CysC_, estimated glomerular filtration rate base on cystatin C; eGFR_Cr-CysC_, estimated glomerular filtration rate base on creatinine and cystatin C; ACR, albumin/creatinine ratio.

The Pearson correlation analysis indicates that mean WSS was positively correlated with the eGFR_Cr_, eGFR_CysC_, and eGFR_Cr-CysC_, and inversely correlated with ACR (all *P* <0.001, Figure 1).

### Effect of peak WSS on renal function

We also assessed the effect of peak WSS on the renal function. Supplementary Table 2 depicts the details of the eGFR_Cr_, eGFR_CysC_, eGFR_Cr-CysC_, and ACR in the four groups classified by the interquartile of the peak WSS. The eGFR_Cr_, eGFR_CysC_, and eGFR_Cr-CysC_ were markedly higher, and the ACR was lower in Q4 than in Q1, Q2, and Q3 (all *P* <0.05). The eGFR_Cr_ and eGFR_Cr-CysC_ were markedly higher, the ACR was lower in Q2 and Q3 than in Q1 (all *P* <0.05). The eGFR_CysC_ was higher in Q3 than in Q1 (*P* <0.05).

Consistent with the mean WSS, the peak WSS was positively correlated with the eGFR_Cr_, eGFR_CysC_, and eGFR_Cr-CysC_, and inversely correlated with ACR (all *P* <0.001, Supplementary Figure 1)

### Carotid WSS independently associated with renal function

To explore the association of mean WSS with renal function, we first adjusted for age and sex in model 1. We found that the mean WSS independently and positively associated with eGFR_Cr_, eGFR_CysC_, and eGFR_Cr-CysC_, and negatively related to ACR (all *P* <0.001). For further identify these associations, we then adjusted for smoking and alcohol intake in model 2, and for body mass index, hypertension, antihypertensive medication, diabetes, lower glucose medication, dyslipidemia, anti-dyslipidemia medication, blood pressure, fasting blood glucose, blood lipids, and carotid artery plaque in model 3. The results showed that the independent associations were still remained (Table 4).

**Table 4:** Regression coefficients (95%) of carotid mean wall shear stress with renal function parameters

	eGFR_Cr_ (ml·min^-1^·1.73m^-2^)		eGFR_CysC_ (ml·min^-1^·1.73m^-2^)		eGFR_Cr-CysC_ (ml·min^-1^·1.73m^-2^)		ACR (mg/g)	
	Beta coefficient (95% C.I.)	*P* value	Beta coefficient (95% C.I.)	*P* value	Beta coefficient (95% C.I.)	*P* value	Beta coefficient (95% C.I.)	*P* value
Model 1^a^	18.696 (15.449, 21.943)	<0.001	30.194 (26.149, 34.239)	<0.001	28.960 (25.288, 32.631)	<0.001	-14.338 (-15.929, -12.748)	<0.001
Model 2^b^	18.972 (15.720, 22.224)	<0.001	30.347 (26.293, 34.402)	<0.001	29.244 (25.568, 32.919)	<0.001	-14.321 (-15.919, -12.724)	<0.001
Model 3^c^	17.840 (14.334, 21.346)	<0.001	23.394 (18.908, 27.880)	<0.001	23.382 (19.677, 27.088)	<0.001	-11.064 (-12.659, -9.469)	<0.001

eGFR_Cr_ indicates estimated glomerular filtration rate base on creatinine; eGFR_CysC_, estimated glomerular filtration rate base on cystatin C; eGFR_Cr-CysC_, estimated glomerular filtration rate base on creatinine and cystatin C; ACR, albumin/creatinine ratio.

^a^Model 1: adjusted for age, sex.

^b^Model 2: model 1 + smoking and alcohol intake.

^c^Model 3: model 2 + body mass index, hypertension, diabetes, dyslipidemia, blood pressure, fasting blood glucose, blood lipids, and carotid artery plaque.

Similar to the mean WSS, the peak WSS was independently and positively associated with eGFR_Cr_, eGFR_CysC_, and eGFR_Cr-CysC_, and negatively related to ACR after adjustment for same confounders included in the models of the mean WSS (Supplementary Table 3).

### Correlations between carotid WSS and biomarkers of endothelial function

We also assessed the correlations of the mean WSS and peak WSS with the FMD, NO, ET-1, ICAM-1, and VCAM-1. We found that mean WSS (Table 5) and peak WSS (Supplementary Table 4) were strong and positively related to FMD and NO, and negatively related to ET-1, ICAM-1, and VCAM-1 after adjustment for confounders in Table 1, including age, sex, smoking, alcohol intake, body mass index, hypertension, antihypertensive medication, diabetes, lower glucose medication, dyslipidemia, anti-dyslipidemia medication, blood pressure, fasting blood glucose, blood lipids, and carotid artery plaque.

**Table 5:** Correlations between carotid mean wall shear stress and biomarkers of endothelial function and inflammation

	Pearson correlation analysis		Partial correlations analysis^a^	
	Correlation coefficient	*P* value	Correlation coefficient	*P* value
Brachial artery flow-mediated dilation, %	0.637	<0.001	0.557	<0.001
Nitric oxide, μmol/L	0.643	<0.001	0.551	<0.001
Endothelin-1, pg/ml	-0.589	<0.001	-0.489	<0.001
Intercellular adhesion molecule-1, ng/ml	-0.465	<0.001	-0.350	<0.001
Vascular cell adhesion molecule-1, ng/ml	-0.428	<0.001	-0.304	<0.001

^a^ adjusted for age, sex, smoking, alcohol intake, body mass index, hypertension, diabetes, dyslipidemia, blood pressure, fasting blood glucose, blood lipids, and carotid artery plaque.

### Correlations between renal function and biomarkers of endothelial function

We also assessed the correlations of the FMD, NO, ET-1, ICAM-1, and VCAM-1 with the eGFR_Cr_, eGFR_CysC_, eGFR_Cr-CysC_, and ACR. We found that FMD and NO were positively related to eGFR_Cr_, eGFR_CysC_, and eGFR_Cr-CysC_, and negatively related to ACR, but ET-1, ICAM-1, and VCAM-1 were negatively related to eGFR_Cr_, eGFR_CysC_, and eGFR_Cr-CysC_, and positively related to ACR even after adjustment for age, sex, smoking, alcohol intake, body mass index, hypertension, antihypertensive medication, diabetes, lower glucose medication, dyslipidemia, anti-dyslipidemia medication, blood pressure, fasting blood glucose, blood lipids, and carotid artery plaque (Table 6).

**Table 6:** Correlations between renal function and biomarkers of endothelial function and inflammation

	eGFR_Cr_ (ml·min^-1^·1.73m^-2^)		eGFR_CysC_ (ml·min^-1^·1.73m^-2^)		eGFR_Cr-CysC_ (ml·min^-1^·1.73m^-2^)		ACR (mg/g)	
	Correlation coefficient	*P* value	Correlation coefficient	*P* value	Correlation coefficient	*P* value	Correlation coefficient	*P* value
A. Pearson correlation analysis								
Brachial artery flow-mediated dilation, %	0.536	<0.001	0.497	<0.001	0.575	<0.001	-0.504	<0.001
Nitric oxide, μmol/L	0.513	<0.001	0.529	<0.001	0.584	<0.001	-0.503	<0.001
Endothelin-1, pg/ml	-0.515	<0.001	-0.486	<0.001	-0.554	<0.001	0.476	<0.001
Intercellular adhesion molecule-1, ng/ml	-0.552	<0.001	-0.417	<0.001	-0.527	<0.001	0.440	<0.001
Vascular cell adhesion molecule-1, ng/ml	-0.400	<0.001	-0.480	<0.001	-0.495	<0.001	0.409	<0.001
B. Partial correlations analysis^a^								
Brachial artery flow-mediated dilation, %	0.380	<0.001	0.344	<0.001	0.429	<0.001	-0.366	<0.001
Nitric oxide, μmol/L	0.350	<0.001	0.374	<0.001	0.432	<0.001	-0.355	<0.001
Endothelin-1, pg/ml	-0.360	<0.001	-0.329	<0.001	-0.400	<0.001	0.326	<0.001
Intercellular adhesion molecule-1, ng/ml	-0.432	<0.001	-0.226	<0.001	-0.363	<0.001	0.264	<0.001
Vascular cell adhesion molecule-1, ng/ml	-0.268	<0.001	-0.339	<0.001	-0.363	<0.001	0.264	<0.001

^a^ adjusted for age, sex, smoking, alcohol intake, body mass index, hypertension, diabetes, dyslipidemia, blood pressure, fasting blood glucose, blood lipids, and carotid artery plaque. eGFR_Cr_ indicates estimated glomerular filtration rate base on creatinine; eGFR_CysC_, estimated glomerular filtration rate base on cystatin C; eGFR_Cr-CysC_, estimated glomerular filtration rate base on creatinine and cystatin C; ACR, albumin/creatinine ratio.

## DISCUSSION

The primary objective of our study was to investigate the correlation of the carotid WSS with renal function in the elderly. The most important findings were those (1) the mean and peak carotid WSS were independently associated with eGFR derived from serum concentrations of creatinine and/or cystatin C using three CKD-EPI (the Chronic Kidney Disease Epidemiology Collaboration) equations and ACR after adjustment for confounders; (2) the carotid WSS was strongly related to endothelial function; and (3) the endothelial function was closely related to renal function. It indicates that rheologic forces may contribute to the crucial effects on renal dysfunction in the elderly. The effects may be mediated by regulation of endothelial function.

In the past decade, as regarding as an important factor of CKD, the hemodynamic has been increasingly emphasized. Verbeke and colleagues [23] reported that brachial artery WSS is significantly lower in patients with end-stage renal disease than in healthy subjects although their brachial artery blood flow is similar. In patients with CKD, the mean and maximum brachial artery wall shear rates were significantly lower than in healthy subjects [25]. Samijo and coworkers [24] assessed WSS in the CCA in end-stage renal failure patients. They found that mean WSS in end-stage renal failure patients who were prior to hemodialysis is significantly lower compared with presumed healthy age- and sex-matched control subjects. However, the studies did not clearly illuminate the relationship between WSS and renal function, and not deeply explore the mechanism of WSS on the renal function.

In the present study, we assessed the mean and peak carotid WSS in 761 community-dwelling elderly using a high-resolution ultrasound. We found that the minimum mean WSS of the right or left CCA was independently and positively correlated with eGFRs that estimated by creatinine and/or cystatin C, and negatively correlated with ACR even after adjustment for confounders. The similar findings were found between peak WSS and eGFRs and ACR. It demonstrates that carotid WSS may play an important role in renal function in the elderly.

It has been well characterized that the endothelial cells response to fluid shear stress [18]. WSS emerges as a major regulator of a large number of endothelial mediators. Under sustained high shear stress, vascular endothelial cells exhibit upregulation of vasodilator NO and down-regulation of vasoconstrictor ET-1, inflammatory adhesion molecules such as ICAM-1 and VCAM-1 [18]. Base on these mechanisms, brachial artery FMD is widely used as a simple and noninvasive clinical indicator of endothelial function [23].

To investigate the mechanism of WSS on the renal function, we investigated the correlations of carotid WSS with endothelial function. We found that the brachial artery FMD and serum NO were significantly higher, and the serum ET-1, ICAM-1, and VCAM-1 were lower in the highest WSS interquartile group than in the lowest WSS interquartile group. After adjusting for confounders, the carotid artery mean WSS and peak WSS were strongly correlated with these endothelial function biomarkers.

In addition, we also investigated the correlations between renal function and endothelial function in the present study. We found that renal function, assessed using eGFR and ACR, was closely related to endothelial function. Therefore, our findings suggested that the effect of carotid WSS on renal function may be mediated by regulation of endothelial function.

In the present study, ACR was used to assess the renal endothelial function as well as renal function. As is known that albuminuria, always represented by ACR, is a marker of renal endothelial dysfunction [27]. Albuminuria appears to link renal and cardiovascular organ damage. Meanwhile, albuminuria has been demonstrated to be strongly associated with systemic endothelial dysfunction in diabetic patients as well as in nondiabetic individuals [28, 29].

NO was used as one of biomarkers of endothelial function in the present study. There is argument about the relationship between NO and renal function. Bahadoran and coworkers [30] have reported that serum NO is independently associated with the risk of CKD in women. Higher level of serum NO metabolites, higher risk of CKD. However, most studies [31–33] demonstrated that NO deficiency contributes to progression of CKD. Similar findings were reported by Wever and colleagues who measured the ^15^N_2_-labeled arginine-to-citrulline conversion using the more direct approach [34]. We found that serum NO level was positively related to eGFRCr, eGFRCysC, and eGFRCr-CysC, and negatively related to ACR.

A strength of our study is that the eGFR was assessed using two new CKD-EPI equations to correct the imprecision of the common serum creatinine-based CKD-EPI equation. One is serum cystatin C-base eGFR equation and the other is cystatin C combined with creatinine-based equation. The precision of the two equations has been validated [35, 36]. In the present study, the eGFRs derived from the three equations were all closely related to carotid WSS. Another strength is that the minimum mean and peak WSS in both sides of CCA were used for analysis. Studies have demonstrated the lower WSS, the more likely to cause various vascular pathologic including impaired endothelial function [37–39].

Several limitations to this study should be considered. First, the study subjects were predominantly made up of Han recruited from the area of Shandong, China. There were essentially geographical limitations and no racial or ethnic minorities. Our findings need to validate in other ethnic groups and in different regions. Second, only CKD-EPI equations were applied to estimate the GFR. It may induce a certain bias. However, the accuracy of CKD-EPI equations has been validated in Chinese population [36]. Third, we did not address the causal relationship between carotid WSS and renal function owing to the cross-sectional nature of the study. Forth, the genetic background was not considered in the present study. Studies reported that genetic variants are significantly related to eGFR [40, 41]. Finally, the renal WSS was not assessed in the present study. There may be differences between the renal WSS and carotid artery WSS.

## MATERIALS AND METHODS

### Study population

From May 2009 to October 2012, a total of 761 older subjects aged 60 years and over [mean and standard deviation (SD): 70.70±6.15 years] were eligible and enrolled from community-dwelling in the Shandong area, China. Among them, 380 were women, and 381 were men. Subjects were excluded if they met any of the following exclusion criteria: end stage of renal disease, hemodialysis, cardio-cerebrovascular events including myocardial infarction and stroke in the previous 3 months, heart failure, secondary hypertension, active malignancy, abnormal liver enzymes (alanine aminotransferase and aspartate aminotransferase >3 times than upper normal range), drug and alcohol abuse, and difficulty with providing informed consent.

This study was conducted in compliance with the “Declaration of Helsinki”. The Research Ethics Committee of the Institute of Basic Medicine, Shandong Academy of Medical Sciences approved this study. Written informed consent was obtained from each subject.

### Ultrasonography of CCA and calculation of WSS

The CCA ultrasound examinations were performed during morning hours in a quiet and temperature-controlled room (20-25 degrees Celsius). The subjects were demanded to discontinue tea, alcohol, caffeine, smoking, anti-histamine, nitrates, and calcium antagonists for 24 h and to fast for 12 h before the examination. After at least a 10-min acclimatization period, the examination was performed using a high-resolution ultrasound with a 7.5-MHz linear array transducer (Vivid *i*, GE Medical Systems Ultrasound Israel Ltd, Tirat Carmel, Israel) and electrocardiogram (ECG) triggering by an experienced ultrasonographer who was blinded to the subjects’ clinical details.

CCA intima-media thickness was measured as the distance form the leading edge of the lumen-initima interface to the collagen-containing upper layer of the adventitia. Carotid artery plaque was defined as a intima-media thickness >1.5 mm.

Accordance with previously described [20], the internal diameters of the CCA at the R (ID_R_) and peak T (ID_T_) waves on the ECG were measured using two-dimensionally guided continuous M-mode tracings. ID_R_ represents the minimum carotid diameter and ID_T_ indicates maximum carotid diameter. Mean velocity (V_M_) and peak systolic velocity (V_PS_), 1-2 cm below the bifurcation, were detected as the mean of three cardiac cycles.

Mean and peak WSS were calculated with the formula [20, 42]:

Mean WSS (Pa) = 8 × η × V_M_/ID_R_

Peak WSS (Pa) = 8 × η × V_PS_/ID_T_

where η is blood viscosity (Pa·s); V is the velocity (m/s); and ID is the lumen diameter (m). Viscosity is equal to 0.0035 Pa·s, as the carotid artery wall is always assumed to be rigid with blood as a Newtonian fluid [43]. Minimum mean and peak WSS in the right or left CCA were used for further analysis.

### Brachial flow-mediated dilation measurement

Brachial artery FMD and CCA ultrasound examinations were performed on the same day. The details of the brachial artery FMD examination have been described in previous studies [44–46]. Briefly, the left brachial artery diameter was measured using a high-resolution ultrasound with a 7.5-MHz linear array transducer (Vivid *i*, GE Medical Systems Ultrasound Israel Ltd, Tirat Carmel, Israel) at rest and during reactive hyperemia. The reactive hyperemia was induced by inflation of a pneumatic tourniquet placed around the forearm to a pressure of 250 mm Hg for 5 min, followed by a release. Arterial diameter was assessed during the end-diastolic phase at a fixed distance from an anatomic marker at baseline and 60, 90, and 120 s after cuff deflation. The maximum diameter from the 3 assessments was used to calculate FMD. The calculation formula is as follows: FMD (%) = [(maximum diameter - baseline diameter)/baseline diameter] × 100 %.

### Serum biomarkers of endothelial function and inflammation measurement

Fasting blood samples were obtained from all subjects and processed within 2 hours. Serum was collected and stored at -80 degrees Celsius until analysis. Serum NO was assessed by the quantification of nitrite using Griess assay [47]. The reagents were purchased from Sigma (St. Louis, MO, USA). Serum ET-1, VCAM-1, and ICAM-1 were measured using enzyme-linked immunosorbent assay (ELISA) kits following the manufacturer's instructions (Bender MedSystems, Vienna, Austria). All samples were tested in duplicate and the mean value was used for further analyze.

### Evaluation of estimated glomerular filtration rate

Renal function was evaluated by an eGFR which calculated from serum creatinine and/or Cystatin C level. Serum creatinine level was detected by the enzymic method (Shanghai Kehua Dongling Diagnostic Products Co., Ltd., China) and cystatin C was measured by the particle-enhanced immunoturbidimetry assay (Beijing Leadman Biomedical Co., Ltd., China) using a Hitachi 7600 automated biochemical analyzer. Three CKD-EPI equations for eGFR [35] were used in the present study:

The equation 1: eGFR_Cr_ (ml·min^-1^·1.73m^-2^) = 141 × min (Scr/κ, 1)^α^ × max (Scr/κ, 1)^-1.209^ × 0.993^Age^ [× 1.018 if female] [× 1.159 if black], where Scr is serum creatinine, κ is 0.7 for females and 0.9 for males, α is -0.329 for females and -0.411 for males, min is the minimum of Scr/κ or 1, and max is the maximum of Scr/κ or 1.

The equation 2: eGFR_CysC_ (ml·min^-1^·1.73m^-2^) = 133 × min (Scys/0.8, 1)^-0.499^ × max (Scys/0.8, 1)^-1.328^ × 0.996^Age^ [× 0.932 if female], where Scys is serum cystatin C, min indicates the minimum of Scys/κ or 1, and max indicates the maximum of Scys/κ or 1.

The equation 3: eGFR_Cr-CysC_ (ml·min^-1^·1.73m^-2^) = 135 × min(Scr/κ, 1) ^α^ × max(Scr/κ, 1)^-0.601^ × min(Scys/0.8, 1)^-0.375^ × max(Scys/0.8, 1)^-0.711^ × 0.995^Age^ [× 0.969 if female] [× 1.08 if black], where Scr is serum creatinine, Scys is serum cystatin C, κ is 0.7 for females and 0.9 for males, α is -0.248 for females and -0.207 for males, min indicates the minimum of Scr/κ or 1, and max indicates the maximum of Scr/κ or 1.

### Evaluation of urinary albumin excretion

Urinary albumin excretion was evaluated on the basis of the urinary ACR. Urinary albumin was tested by morning first void sterile urinary spot samples using immunonephelometry.

### Statistical analysis

All statistical analyses were carried out using the SPSS for Windows software package, version 22.0 (SPSS Inc., Chicago, IL, USA). Continuous data were expressed as mean ±SD or as median with interquartile range (IQR, 25th and 75th percentiles) depending on the normality of the data. The normality was determined using the Kolmogorov–Smirnov test. Categorical data were expressed as numbers (percentages). Subjects were grouped by the interquartiles of either the mean WSS or peak WSS. The differences in continuous variables among the groups were assessed using one-way analysis of variance (ANOVA) with the Bonferroni procedure or the Kruskal–Wallis test with Wilcoxon rank-sum test. The differences in categorical variables among the groups were assessed using the chi-square test. Pearson correlation coefficients were used to assess the relationships of the mean WSS and peak WSS to eGFR_Cr_, eGFR_CysC_, eGFR_Cr-CysC_, and ACR. Partial correlations analysis was used to determined the relationships of the mean WSS and peak WSS to FMD, NO, ET-1, ICAM1, and VCAM1, and the relationships of the FMD, NO, ET-1, ICAM1, and VCAM1 to eGFR_Cr_, eGFR_CysC_, eGFR_Cr-CysC_, and ACR. A multiple linear regression analysis was performed to determine if any factors were independently associated with renal function. Mean WSS and peak WSS were included as an independent factor, respectively, in the regression models. Model 1 was adjusted for age, sex. Model 2 was further adjusted for smoking and alcohol intake, and model 3 was further adjusted for body mass index, hypertension, antihypertensive medication, diabetes, lower glucose medication, dyslipidemia, anti-dyslipidemia medication, blood pressure, fasting blood glucose, and blood lipids. A *P*-value of <0.05 was considered to be significant.

## CONCLUSIONS

In conclusion, there is an independent correlation of carotid WSS with renal function in the elderly. The rheologic forces may play an important role in renal function changing. The correlation may be mediated by regulation of endothelial function. The multi-racial, multi-ethnic, multi-geographic, and longitudinal studies are needed to clarify the role of carotid WSS in CKD.

## SUPPLEMENTARY FIGURE AND TABLES



## Abbreviations

CKD: chronic kidney disease
CCA: common carotid artery
WSS: wall shear stress
eGFR: estimated glomerular filtration rate
ACR: albumin/creatinine ratio
Cr: creatinine
Cys C: cystatin C
UA: urinary albumin
FMD: flow-mediated dilation
NO: nitric oxide
ET-1: endothelin-1
ICAM-1: intercellular adhesion molecule-1
VCAM-1: vascular cell adhesion molecule-1
CKD-EPI: the Chronic Kidney Disease Epidemiology Collaboration.
