# Supplementary material for: Carotid artery wall shear stress is independently correlated with renal function in the elderly
Source: Oncotarget. 2018 Jan 2;9(4):5251–62. doi: 10.18632/oncotarget.23825 (PMC5797047; doi:10.18632/oncotarget.23825)
Supplement: Supplementary file 1 [file oncotarget-09-5251-s001.pdf]

Carotid artery wall shear stress is independently correlated with renal function in the elderly

SUPPLEMENTARY MATERIALS

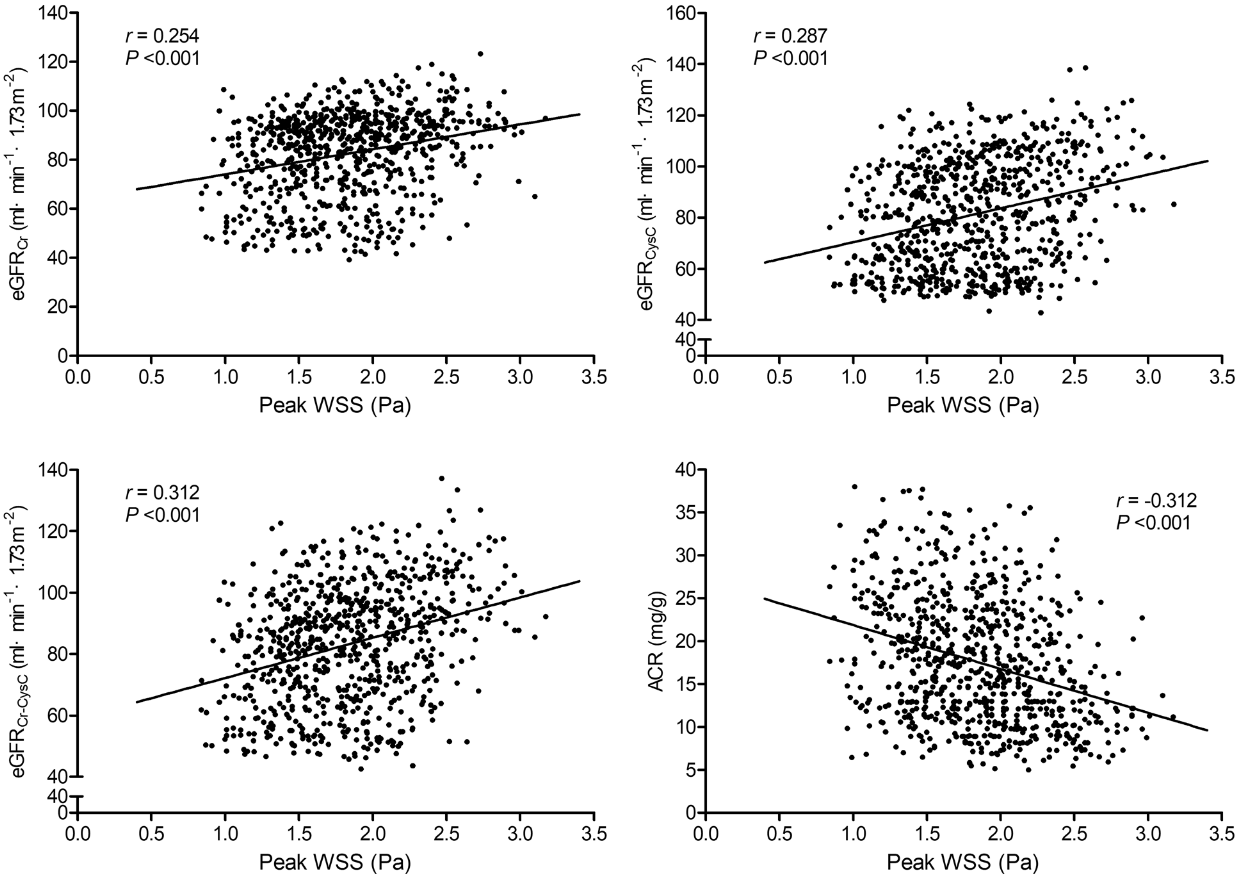

Supplementary Figure 1: Correlations of carotid peak wall shear stress with the eGFR$_{Cr}$, eGFR$_{CysC}$, eGFR$_{Cr-CysC}$, and ACR. WSS indicates wall shear stress; eGFR$_{Cr}$, estimated glomerular filtration rate base on creatinine; eGFR$_{CysC}$, estimated glomerular filtration rate base on cystatin C; eGFR$_{Cr-CysC}$, estimated glomerular filtration rate base on creatinine and cystatin C; ACR, albumin/creatinine ratio.

Supplementary Table 1: Clinical and biochemical characteristics of participants grouped by the interquartile of carotid peak wall shear stress

	Q1 (*n*=191)	Q2 (*n*=190)	Q3 (*n*=190)	Q4 (*n*=190)	*P* value
Clinical parameters					
Age, years	71.51±6.38	70.63±6.27	71.24±5.86	69.42±5.90*	0.004
Sex, Male:Female	89:102	100:90	97:93	95:95	0.684
BMI, kg/m²	24.28±2.71	24.34±3.05	24.24±2.92	24.40±2.78	0.954
SBP, mm Hg	145.72±12.42	141.05±12.74*	140.49±13.70*	138.05±14.85*	<0.001
DBP, mm Hg	69.62±7.23	70.38±6.95	69.70±6.59	69.22±7.41	0.445
Biochemical parameters					
TCHO, mmol/L	4.43±0.62	4.60±0.69	4.64±0.65*	4.61±0.64*	0.008
TG, mmol/L	1.43±0.37	1.42±0.34	1.47±0.34	1.49±0.32	0.157
HDL-c, mmol/L	1.15±0.16	1.13±0.17	1.14±0.19	1.14±0.20	0.740
LDL-c, mmol/L	2.64±0.59	2.83±0.68*	2.83±0.64*	2.79±0.59	0.006
FPG, mmol/L	5.26±0.83	5.38±0.99	5.31±0.98	5.35±0.85	0.601
Cr, mg/dl	0.82 (0.70, 0.98)	0.78 (0.66, 0.93)	0.79 (0.66, 0.93)	0.71 (0.61, 0.84)*,†,‡	<0.001
Cys C, mg/L	0.99±0.19	0.94±0.21	0.94±0.21	0.85±0.22*,†,‡	<0.001
UA, mg/L	1.67 (0.98, 2.37)	1.35 (0.90, 1.97)	1.30 (0.80, 2.00)*	0.92 (0.68, 1.41)*,†,‡	<0.001
Covariates					
Current smoker, *n* (%)	41 (21.47)	50 (26.32)	47 (24.74)	40 (21.05)	0.557
Alcohol consumption, *n* (%)	69 (36.13)	79 (41.58)	77 (40.53)	84 (44.21)	0.442
Hypertension, *n* (%)	155 (81.15)	114 (60.00)*	117 (61.58)*	103 (54.21)*	<0.001
Antihypertension, *n* (%)	150 (96.77)	107 (93.86)	104 (88.89)*	83 (80.58)*,†	<0.001
Diabetes, *n* (%)	18 (9.47)	25 (13.16)	25 (13.16)	25 (13.16)	0.602
Antidiabetes, *n* (%)	18 (100.00)	23 (92.00)	22 (88.00)	24 (96.00)	0.413
Dyslipidemia, *n* (%)	57 (29.84)	43 (22.63)	57 (30.00)	63 (33.16)†	0.003
Anti-dyslipidemia, *n* (%)	28 (49.12)	21 (48.84)	32 (56.14)	27 (42.86)	0.907
Carotid plaque, *n* (%)	97 (50.79)	69 (36.32)*	55 (28.95)*	41 (21.58)*,†	<0.001
Endothelial function parameters					
Brachial artery FMD, %	10.35±2.71	11.14±2.83*	12.13±2.98*,†	13.77±2.47*,†,‡	<0.001
NO, μmol/L	55.48±12.70	61.14±13.69*	64.22±13.67*	71.50±13.18*,†,‡	<0.001
ET-1, pg/ml	45.65±8.01	44.11±9.33	41.70±9.20*,†	37.62±7.69*,†,‡	<0.001
ICAM-1, ng/ml	152.57±35.72	145.50±37.77	136.53±33.66*	126.91±28.20*,†,‡	<0.001
VCAM-1, ng/ml	6551.93 (567.57, 739.62)	621.25 (552.23,705.62)	613.58 (559.90, 697.85)	582.91 (500.71, 659.60)*,†,‡	<0.001

Data are mean±standard deviation or number (percentage). Q1 indicates the first interquartile; Q2, the second interquartile; Q3, the third interquartile; Q4, the forth interquartile; BMI, body mass index; SBP, systolic blood pressure; DBP, diastolic blood pressure; TCHO, total cholesterol; TG, triglycerides; HDL-c, high-density lipoprotein cholesterol; LDL-c, low-density lipoprotein cholesterol; Cr, creatinine; Cys C, cystatin C; UA, Urinary albumin; FMD, flow-mediated dilation; NO, nitric oxide; ET-1, endothelin-1; ICAM-1, intercellular adhesion molecule-1; and VCAM-1, vascular cell adhesion molecule-1. *P <0.05, as compared to Q1; †P <0.05, as compared to Q2; ‡P <0.05, as compared to Q3.

Supplementary Table 2: Estimated glomerular filtration rates and albumin/creatinine ratio of participants grouped by the interquartile of carotid peak wall shear stress

	Q1 (*n*=191)	Q2 (*n*=190)	Q3 (*n*=190)	Q4 (*n*=190)	*P* value
$eGFR_{Cr}$, ml/min/1.73 m^{-2}	77.06±17.52	81.95±16.67*	82.07±18.12*	89.02±15.49*,†,‡	<0.001
$eGFR_{CysC}$, $ml \cdot min^{-1} \cdot 1.73m^{-2}$	75.12±18.47	80.23±19.96	80.92±20.73*	89.68±21.29*,†,‡	<0.001
$eGFR_{Cr\text{-}CysC}$, $ml \cdot min^{-1} \cdot 1.73m^{-2}$	76.56±17.17	82.12±17.95*	82.58±19.29*	91.45±18.45*,†,‡	<0.001
ACR, mg/g	20.21±7.61	18.40±7.13*	17.31±6.83*	14.42±7.25*,†,‡	<0.001

Data are mean±standard deviation. Q1 indicates the first interquartile; Q2, the second interquartile; Q3, the third interquartile; Q4, the forth interquartile; $eGFR_{Cr}$, estimated glomerular filtration rate base on creatinine; $eGFR_{CysC}$, estimated glomerular filtration rate base on cystatin C; $eGFR_{Cr\text{-}CysC}$, estimated glomerular filtration rate base on creatinine and cystatin C; ACR, albumin/creatinine ratio. **P* <0.05, as compared to Q1; †*P* <0.05, as compared to Q2; ‡*P* <0.05, as compared to Q3.

Supplementary Table 3: Regression coefficients (95%) of carotid peak WSS with renal function parameters

	$eGFR_{Cr}$ ($ml \cdot min^{-1} \cdot 1.73m^{-2}$)		$eGFR_{CysC}$ ($ml \cdot min^{-1} \cdot 1.73m^{-2}$)		$eGFR_{Cr-CysC}$ ($ml \cdot min^{-1} \cdot 1.73m^{-2}$)		ACR (mg/g)	
	Beta coefficient (95% C.I.)	*P* value	Beta coefficient (95% C.I.)	*P* value	Beta coefficient (95% C.I.)	*P* value	Beta coefficient (95% C.I.)	*P* value
Model 1[a]	5.645 (3.534, 7.755)	<0.001	9.540 (6.824, 12.257)	<0.001	9.443 (6.959, 11.926)	<0.001	-4.669 (-5.774, -3.564)	<0.001
Model 2[b]	5.826 (3.707, 7.946)	<0.001	9.561 (6.860, 12.321)	<0.001	9.608 (7.113, 12.103)	<0.001	-4.637 (-5.748, -3.525)	<0.001
Model 3[c]	6.007 (3.868, 8.147)	<0.001	7.861 (5.118, 10.604)	<0.001	7.946 (5.636, 10.256)	<0.001	-3.536 (-4.547, -2.525)	<0.001

$eGFR_{Cr}$ indicates estimated glomerular filtration rate base on creatinine; $eGFR_{CysC}$, estimated glomerular filtration rate base on cystatin C; $eGFR_{Cr-CysC}$, estimated glomerular filtration rate base on creatinine and cystatin C; ACR, albumin/creatinine ratio.

[a]Model 1: adjusted for age, sex.

[b]Model 2: model 1 + smoking and alcohol intake.

[c]Model 3: model 2 + body mass index, hypertension, diabetes, dyslipidemia, blood pressure, fasting blood glucose, blood lipids, and carotid artery plaque.

Supplementary Table 4: Correlations between carotid peak WSS and biomarkers of endothelial function and inflammation

	Pearson correlation analysis		Partial correlations analysis[a]	
	correlation coefficient	*P* value	correlation coefficient	*P* value
Brachial artery flow-mediated dilation, %	0.457	<0.001	0.401	<0.001
Nitric oxide, μmol/L	0.425	<0.001	0.353	<0.001
Endothelin-1, pg/ml	-0.372	<0.001	-0.304	<0.001
Intercellular adhesion molecule-1, ng/ml	-0.290	<0.001	-0.222	<0.001
Vascular cell adhesion molecule-1, ng/ml	-0.228	<0.001	-0.148	<0.001

[a] adjusted for age, sex, smoking, alcohol intake, body mass index, hypertension, diabetes, dyslipidemia, blood pressure, fasting blood glucose, blood lipids, and carotid artery plaque.
